# Supplementary material for: Chromothripsis during telomere crisis is independent of NHEJ, and consistent with a replicative origin
Source: Genome Res. 2019 May;29(5):737–49. doi: 10.1101/gr.240705.118 (PMC6499312; doi:10.1101/gr.240705.118)
Supplement: Supplemental Material [file supp_gr.240705.118_Supplemental_file_1.zip › contigs/annotated_contigs/DB82/contig.2.DB82_length_243_mean_cov_3.7037037037.docx]

**DB82_length_243_mean_cov_3.7037037037**

```
AGGATTCATGGACAAGAATGGGTGATAAATGTCCCAGATACCTTGCCCTTTCACTGAGATGACT|C|AGTGTAAGGAAGGGGGGCTTCA
 >chr5:174736253-174736318 + E=7e-25                             >chr5:174748907-174749
AACAGGAGGCACAGACTCCTTAGAGATGCCTCTTGCTGCCAATCACCTCTCCTCTACCCTTGACCAGGAAACCTCTTCAGAAACTCACA
086 + E=8e-94
GAATGACAGGCGGTCAGCATACACCTTCTCTATGTGCAGAGAAGGGGACAGGTCCCACTGTGGCATT
```
